# Genetic Analysis of the Conserved Population of Dengchuan Cattle Based on High Concordance SNP loci

**DOI:** 10.3390/ani15202937

**Published:** 2025-10-10

**Authors:** Jiangyu Long, Jingjing Su, Shiyan Sui, Huimin Li, Rong Jiang, Linjie Xu, You Tan, Birong Zhang

**Affiliations:** 1 College of Public Health, Dali University, Dali 671000, China; 20242011060009@stu.dali.edu.cn (J.L.); 19869022931@163.com (R.J.); 15887947743@163.com (L.X.); xiat1108@gmail.com (Y.T.); 15125255302@163.com (B.Z.); 2 Management Department of Laboratory, Dali University, Dali 671000, China; 911796@dali.edu.cn; 3 Yunnan Provincial Key Laboratory of Entomological Biopharmaceutical R&D, College of Pharmacy, Dali University, Dali 671000, China; 4 Dali Dairy Association, Dali 671000, China; lhm11186@126.com

**Keywords:** SNP, Dengchuan cattle, purity identification, genetic diversity, PCA

## Abstract

**Simple Summary:**

Dengchuan cattle are a rare local dairy breed from Southwest China, famous for their high milk fat content and cultural importance. Unfortunately, their numbers have declined sharply because of uncontrolled crossbreeding with foreign cattle. This study set out to investigate the genetic status of the remaining population and to provide tools for their conservation. Using a set of genetic markers called single-nucleotide polymorphisms, we examined the genetic variation of 74 animals. The results showed that the cattle still maintain a moderate level of genetic diversity, which means there is some variety left in their genes. At the same time, we found that the number of breeding animals that effectively contribute to the next generation is very low, and some family lines are over-represented. This situation may cause a loss of genetic variation in the future. Importantly, we identified 61 genetic markers that can be used to check the purity of Dengchuan cattle. These markers will help breeders to maintain the unique features of this valuable breed. Our findings highlight the urgent need to carefully manage and protect Dengchuan cattle so that they can continue to serve as an important resource for future farming and cultural heritage.

**Abstract:**

Local livestock genetic resources are crucial for sustainable agriculture and biodiversity conservation. Dengchuan cattle, a nationally protected dairy breed in China, are esteemed for their high milk fat content and cultural significance. However, they have been threatened by crossbreeding with exotic high-yielding breeds, resulting in a decline in purebred resources. In this study, we evaluated the genetic diversity and structure of a conserved population using 100K SNP microarray data from 74 individuals. After implementing strict quality control measures, 78,460 loci were retained for principal component analysis (PCA), which identified 100 SNPs most associated with PC1. After calculating high-consistency loci using PLINK, based on allelic consistency, we selected 61 high-stability markers to represent 60 individuals for further analysis. Genetic diversity parameters indicated moderate polymorphism, with an effective population size (Ne) of 2.293, observed heterozygosity (Ho) of 0.300, expected heterozygosity (He) of 0.326, and an average polymorphic information content (PIC) of 0.261. A paired t-test confirmed a highly significant difference between Ho and He (*p* < 0.001). Runs of homozygosity (ROH) revealed a moderate level of inbreeding (F_ROH_ = 0.0928), with bulls exhibiting slightly higher values than females. Neighbor-joining (NJ) clustering further indicated clear lineage distinctions among bulls, but lower kinship among females. Overall, Dengchuan cattle exhibit moderate genetic diversity but face risks due to a small Ne and an unbalanced family structure. Targeted breeding strategies and genetic monitoring are recommended to ensure sustainable conservation and utilization.

## 1. Introduction

Local livestock and poultry genetic resources serve as the foundation for the stable development of animal husbandry. They possess irreplaceable strategic value in enhancing the territorial adaptability of agricultural production and safeguarding the unique biogenetic gene pool [1]. However, the rapid expansion of intensive farming practices and the high dependence on a limited number of high-yielding commercial breeds have led to a significant loss of genetic diversity among local breeds globally. Consequently, many invaluable genetic resources are now on the brink of extinction [2]. As the country with the richest livestock and poultry genetic resources in the world, China holds a unique position globally regarding its local domestic cattle resources. The population of yellow cattle in China comprises two major genealogical lineages: Bos taurus and Bos indicus. These lineages entered China through various migration routes during different historical periods. Following an extensive period of crossbreeding and ecological adaptation, the population has evolved, resulting in a rich genetic diversity [3].

Among the various local yellow cattle breeds, Dengchuan cattle stand out as a highly representative special breed. This breed is primarily found in Eryuan County, Dali Prefecture, Yunnan Province, and is recognized as a nationally protected breed, having been included in the National List of Livestock Genetic Resources Breeds. As a rare local yellow cattle breed primarily noted for its dairy performance in China, Dengchuan cattle are distinguished by their high milk fat ratio of 6.89%. This characteristic renders them a valuable raw material for traditional high-value-added dairy products, thus holding significant potential for economic development and cultural value. However, the construction of the ‘Southern Dairy Capital’ in Eryuan County has led to the introduction of a substantial number of high-yielding breeds, such as Holstein. The absence of scientific guidance on crossbreeding and improvement has resulted in a drastic decline in the population of purebred Dengchuan cattle, leading to severe erosion of the local gene pool and placing this breed on the verge of extinction [4]. Dengchuan cattle are facing a predicament where increased industrial development leads to the endangerment of this breed. This situation underscores the profound contradiction between the protection of local genetic resources and the development of modern agricultural monocultures.

Currently, there is no systematic study that comprehensively assesses the genetic diversity, inbreeding levels, and population structure of the existing Dengchuan cattle population. In the absence of critical genomic information, implementing scientifically effective breeding conservation measures is challenging. Modern genomic technologies, particularly high-throughput single-nucleotide polymorphism (SNP) microarrays and whole-genome resequencing, have been extensively utilized to evaluate genetic health, elucidate individual relatedness, and inform scientific breeding selection [5]. High-consistency SNP loci are frequently subjected to evolutionary conservative selection, possess significant functional or adaptive importance, and serve as population-specific markers. These markers are crucial for elucidating varietal differences and ancestral components [6].

Principal component analysis (PCA) is a crucial tool for analyzing population genetic structure. The first principal component (PC1) signifies the direction of maximum variance in the data and typically highlights the most significant genetic differences among populations [7]. In this study, we identified the top 100 SNP loci contributing most significantly in the PC1 direction using PCA, and further selected those that also met the criteria for high concordance as calculated by PLINK. We then combined these loci with allele frequency data to conduct preliminary quality control of the Dengchuan cattle population. This approach allowed us to obtain representative individuals with relatively clear genetic backgrounds and high credibility, which served as the foundation for subsequent genetic analyses. Thus, conducting a systematic genomic assessment of the Dengchuan cattle conservation populations has become an urgent task. Utilizing SNP microarray data, this study aims to elucidate the levels of genetic diversity, inbreeding status, and population structure of the existing Dengchuan cattle population, thereby providing a scientific basis and technical support for the salvage conservation of this breed and the sustainable use of its genetic resources.

## 2. Materials and Methods

### 2.1. Experimental Animals

In this study, blood samples were collected from 74 Dengchuan cattle (12 bulls and 62 females) on 21 November 2024, at the location east of Dengchuan Cattle Conservation Farm (near the south side of S310, with GPS coordinates approximately 26.107° N, 99.956° E) in Eryuan County, Yunnan Province. The samples were placed in 15 mL centrifuge tubes and stored at −80 °C for subsequent use.

### 2.2. Experimental Methods

#### 2.2.1. Genomic DNA Extraction and Quality Testing of Blood Samples

The collected blood samples were stored in EDTA K_2_ blood collection tubes, and blood sample collection cards were utilized concurrently. Subsequently, the samples were transferred to Nuqin Biological Company for DNA extraction and quality assessment. Genomic DNA (gDNA) was extracted from the blood samples of Dengchuan cattle using the magnetic bead method. The integrity, purity (OD_260_ₙₘ/OD_280_ₙₘ), and concentration of the DNA were evaluated using an ultraviolet spectrophotometer (NanoDrop 2000, Thermo Fisher Scientific, Shanghai, China).

#### 2.2.2. SNP Typing and Quality Control

SNP genotyping was conducted using the Illumina 100K SNP BeadChip (Illumina, Shanghai, China) [8], manufactured by Illumina, USA. Quality control of samples and SNP loci was performed using Plink software (version 1.90) [9]. SNP loci that did not meet the specified quality control criteria were excluded from the analysis. Specifically, only loci located on autosomes were retained, while those with a SNP detection rate or individual detection rate below 90%, a Hardy–Weinberg *p*-value less than 1 × 10^−6^, and a Minor Allele Frequency (MAF) below 0.01 were discarded.

#### 2.2.3. Purity Identification and Screening

Initially, the raw SNP data files were imported into DNA extraction and quality eigenvalue and eigenvalue format files. Subsequently, PCA was performed using R to calculate the contribution of each SNP locus to PC1. The PLINK software was then utilized to calculate the allelic concordance and frequency of each SNP locus, with the Biostrings [10] package in R further verifying the concordance and base frequency. Following this, Notepad++ (V8.8.3.0) was employed to transpose and combine the MAP format file to identify the primary allele (the allele with the highest frequency at a specific SNP locus) and secondary allele (the allele with a lower frequency than the primary allele at that SNP locus) of each locus. Loci with a primary allele frequency exceeding 80% were classified as high concordance. Based on these high concordance loci, the Dengchuan cattle population was organized, and specific individuals were selected for further analysis.

#### 2.2.4. Genetic Diversity Analysis of Dengchuan Cattle Conservation Populations

The effective population size (Ne) of the Dengchuan cattle conservation population was analyzed using Plink in conjunction with R(V4.5.1) software. For this population, we computed the expected heterozygosity (He), observed heterozygosity (Ho), polymorphism information content (PIC), minor allele frequency (MAF), Shannon’s information index (SHI), and the inbreeding coefficient (F) utilizing Plink (V1.90). Based on the calculated MAF, the proportion of polymorphic markers (PN) was determined using R software. All values are presented as mean ± standard deviation (SD) unless otherwise stated.

#### 2.2.5. Kinship Analysis of Dengchuan Cattle Conservation Groups

Using GCTA (V1.94) [11] software, we calculated the kinship coefficients among individuals of Dengchuan cattle and constructed the genomic kinship matrix (G matrix). These kinship analysis results were then visualized using R software. Additionally, we employed Plink to generate the Identity by State (IBS) distance matrix, and the corresponding genetic distance analysis was also visualized in R. Furthermore, Runs of Homozygosity (ROH) were identified for each sample using Plink. The resulting output was subsequently processed in R to calculate the genomic inbreeding coefficient (F_ROH_), which was visualized as a violin plot.

#### 2.2.6. Family Structure Analysis of Dengchuan Cattle Breeding Groups

To analyze the family structure of the conserved population of Dengchuan cattle, the bull samples were first clustered using the Neighbor-Joining (NJ) method in IQ-TREE [12] Family classification was then based on genomic kinship: first, bulls with a kinship coefficient ≥ 0.1 were defined as a core family, and subsequently, cows were assigned to that family if their kinship coefficient with any of its bulls was also ≥ 0.1.

## 3. Results

### 3.1. DNA Detection

In this study, a total of 74 DNA samples were extracted from Dengchuan cattle. The integrity of the extracted DNA samples was satisfactory. Additionally, the average concentration and OD_260_ nm/OD_280_ nm ratios of all DNA samples met the requirements for microarray detection, making them suitable for subsequent analysis.

### 3.2. SNP Typing and Quality Control of 74 Dengchuan Cattle

Genome-wide single-nucleotide polymorphisms (SNPs) were identified using bovine 100K SNP microarrays, resulting in the acquisition of 95,256 SNP markers. SNP loci that did not meet the established quality control criteria were excluded. Specifically, only autosomal loci were retained, and SNPs were discarded if they had a detection rate or individual detection rate below 90%, a Hardy–Weinberg *p*-value less than 1 × 10^−6^, or a Minor Allele Frequency (MAF) below 0.01. Following these quality control measures, 78,460 SNPs remained for further analysis (Table 1).

### 3.3. Results of Purity Identification and Screening

**Figure 1:**
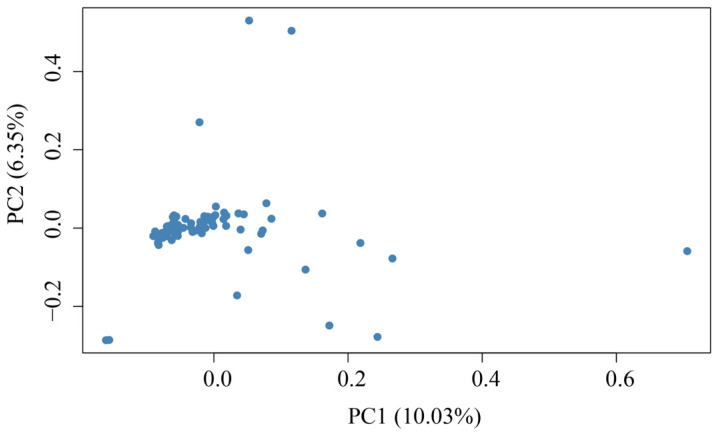
PCAs of 74 Dengchuan cattle.

The SNP data from Dengchuan cattle were subjected to PCA, as illustrated in Figure 1. From this analysis, the top 100 loci contributing significantly in the PC1 direction were identified, as presented in Supplementary Table S3. A total of 78,460 loci were subsequently evaluated for consistency using PLINK, based on the previously defined criteria for high consistency loci: loci with less than 30% consistency are classified as low consistency, those with consistency between 30% and 80% are classified as medium consistency, and loci with greater than 80% consistency are classified as high consistency. Ultimately, 52,584 loci were identified as high consistency loci via PLINK. From the 100 high-contributing loci in the PC1 direction, those that also met the high consistency criteria (identified by PLINK) were selected, resulting in 61 loci retained, as shown in Supplementary Table S4. The Dengchuan cattle population was then ranked according to the number of primary alleles present at these 61 loci, leading to the selection of the top 60 Dengchuan cattle, detailed in Supplementary Table S5.

### 3.4. SNP Typing and Quality Control of 60 Dengchuan Cattle

**Figure 2:**
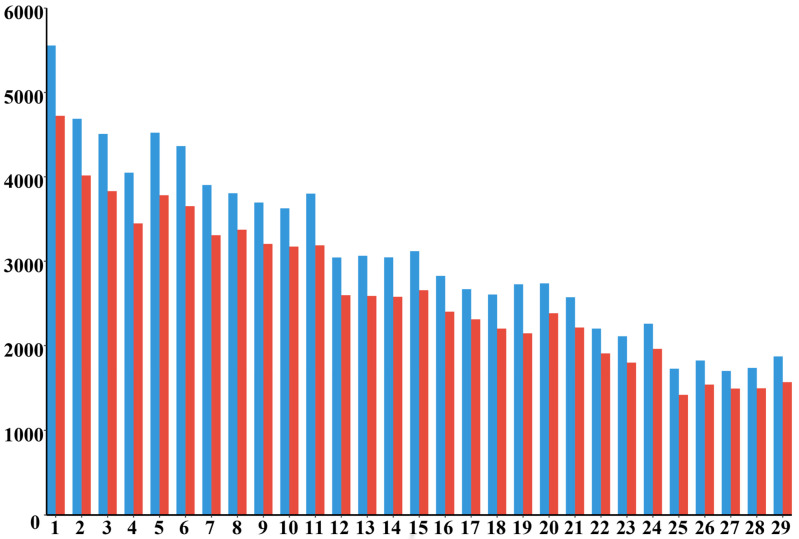
Comparison of SNP numbers on each autosomal chromosome before and after quality control. The *x*-axis represents chromosome numbers, and the *y*-axis indicates the number of SNPs. Blue bars show SNP counts before quality control, and red bars show SNP counts after quality control.

The raw SNP data of the remaining 60 Dengchuan cattle were analyzed using Plink software, yielding 95,256 SNP markers. After quality control, 76,946 high-quality autosomal SNPs (80.8% of the initial set) remained for subsequent analyses of genetic diversity, inbreeding, relatedness, and family lineage construction (Table 2). Quality control removed in total 18,310 markers (19.2%): 4907 (5.15%) markers located on sex chromosomes or unplaced scaffolds (X, Y, 0), 8242 (8.65%) markers with call rate (detection rate) < 0.90, 50 (0.05%) markers with Hardy–Weinberg equilibrium *p* < 1 × 10^−6^, and 5111 (5.36%) markers with minor allele frequency (MAF) < 0.01. Figure 2 compares the SNP counts per autosome before and after filtering; the post-Quality Control counts retain the same descending pattern from larger to smaller chromosomes, and no autosome shows disproportionate SNP loss, indicating that filtering steps acted uniformly across the genome.

### 3.5. Genetic Diversity Analysis of Dengchuan Cattle Breeding Population

**Figure 3:**
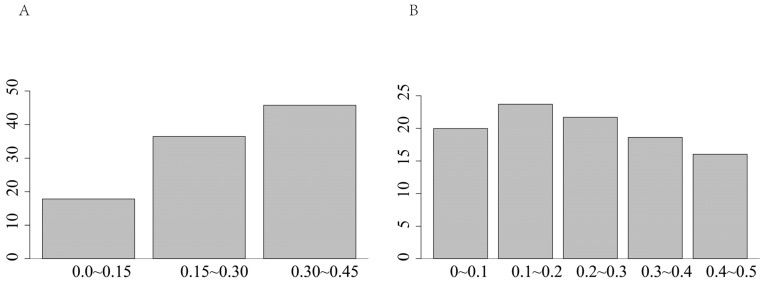
Distribution of MAF and PIC in Dengchuan cattle. (**A**) Distribution of minor allele frequency (MAF), where the *x*-axis represents allele frequency intervals and the *y*-axis indicates the proportion of the population. (**B**) Distribution of polymorphic information content (PIC), where the *x*-axis corresponds to PIC intervals and the *y*-axis indicates the proportion of the population.

**Figure 4:**
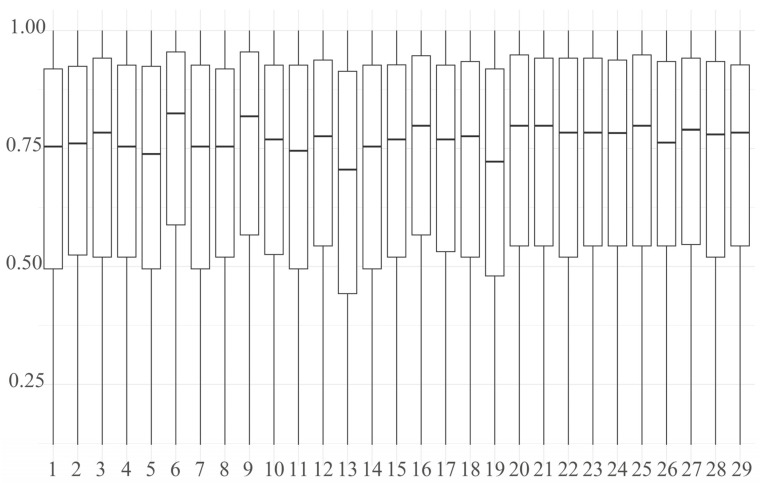
Distribution of Shannon’s information index (SHI) across autosomes. The *x*-axis corresponds to chromosomes, and the *y*-axis represents SHI values.

The genetic diversity analysis revealed a low effective population size (Ne = 2.29) for the Dengchuan cattle conservation population. The observed heterozygosity (Ho = 0.300) was significantly lower than the expected heterozygosity (He = 0.326), with the difference being statistically significant (paired t-test, *p* < 0.001), indicating a notable deficit of heterozygotes. This heterozygote deficit is consistent with the presence of inbreeding within the population. To further investigate this, individual inbreeding coefficients (F) were calculated, ranging from –0.193 to 0.384. The mean F was 0.071 ± 0.122(SD), which was statistically significant and greater than zero (one-sample t-test, *p* < 0.01). This confirms a low but significant level of inbreeding across the population, likely resulting from the small population size and historical lineage duplication. The distribution of polymorphic information content (PIC) classes (Figure 3A) yielded a mean PIC of 0.261 ± 0.101(SD). The minor allele frequency (MAF) spectrum (Figure 3B) showed that the 0.10–0.20 interval had the highest proportion of loci (23.74%), whereas the 0.40–0.50 interval was the lowest (16.02%). Shannon’s diversity index (SHI) across chromosomes ranged from 0.12 to 1.00 with an average of 0.707 (Figure 4).

### 3.6. IBS and Genomic Relationship (G) Matrix Analyses of the Dengchuan Conservation Population

**Figure 5:**
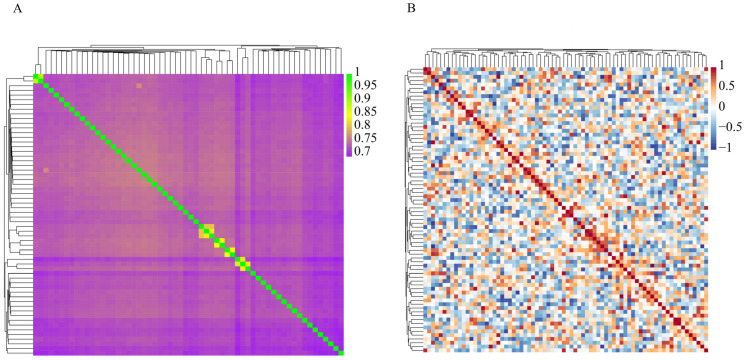
Genomic distance and relationship matrices of the Dengchuan cattle conservation herd. (**A**) Identity-by-State (IBS) distance matrix. (**B**) Genomic relationship matrix (G-matrix).

Pairwise identity-by-state (IBS) distances among the 60 Dengchuan cattle ranged from 0.108 to 0.312 (mean 0.272 ± 0.019 SD). Bulls showed a slightly higher average IBS distance (0.297 ± 0.022 SD), indicating that the sires retained in the conservation group are comparatively divergent—an advantageous feature for managing future matings. The IBS heatmap with hierarchical clustering (Figure 5A) reveals that most pairwise relationships fall in a moderate distance band, while only a few compact blocks of lower distance identify small clusters of more closely related individuals (putative half-sib or family subgroups).

The genomic relationship (G) matrix (Figure 5B) corroborates this pattern: off-diagonal genomic relationship coefficients are predominantly low to moderate, and high-value patches are sparse and localized. Diagonal elements equal 1 by definition. The absence of extensive contiguous high-relatedness blocks suggests no widespread recent inbreeding, consistent with the near-zero to slightly positive inbreeding coefficients (F) and the moderate F_ROH_ values reported above. Together, the IBS and G matrices indicate an overall moderate genetic distance structure with a limited number of kinship clusters that can be monitored to avoid mating within clusters and to preserve genetic diversity.

### 3.7. ROH-Based Inbreeding Coefficient Analysis of Dengchuan Cattle Conservation Population

**Figure 6:**
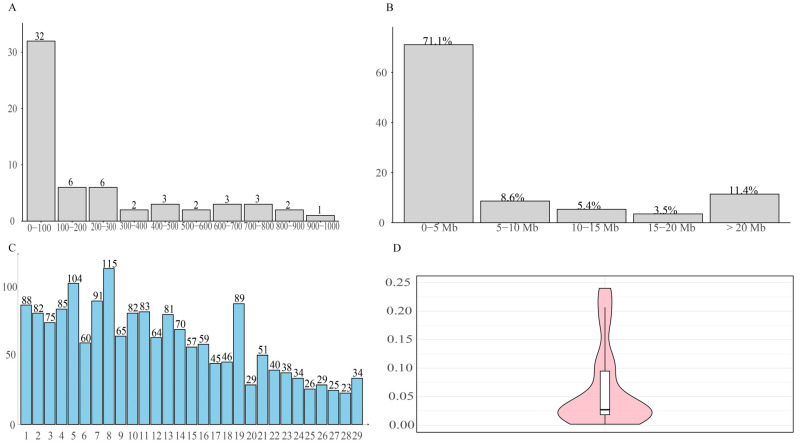
Distribution of ROH in Dengchuan cattle. (**A**) Distribution of individual ROH lengths, where the *x*-axis represents the length of ROH (Mb), and the *y*-axis shows the proportion of the total number of ROH. (**B**) Distribution of the total ROH length per individual, with the *x*-axis indicating ROH length categories and the *y*-axis representing the number of individuals. (**C**) Distribution of the number of ROH across chromosomes, where the *x*-axis corresponds to chromosome numbers and the *y*-axis to the number of ROH on each chromosome. (**D**) Distribution of inbreeding coefficients (FROH) based on ROH, where the boxes indicate the interquartile range, and the violin shape represents the probability density distribution of ROH.

The ROH analysis identified 1770 runs across the 60 Dengchuan cattle, averaging 29.5 ± 12.4 (SD)ROH segments per individual (range 3–65) (Figure 6A–D). Individual total ROH length ranged from 5.10 to 916.36 Mb (mean 232.83 ± 264.60(SD) Mb); 32 animals (53.33%) had < 100 Mb, whereas only one animal fell in the 900–1000 Mb class (Figure 6B). Single-segment lengths were dominated by short ROH (0–5 Mb; 71.1%), with progressively fewer segments in longer classes; the 15–20 Mb class accounted for 3.50% and very long segments (>20 Mb) were rare (Figure 6A). The chromosomal distribution was uneven: chromosome 8 harbored the highest number of ROH (115), while chromosome 28 showed the lowest count (23) (Figure 6C). Genomic inbreeding based on ROH (F_ROH_) was generally low to moderate (mean 0.093 ± 0.105(SD); bulls 0.119 ± 0.107(SD); females 0.087 ± 0.105(SD)), with 37 individuals (61.7%) in the 0–0.06 class (Figure 6D). The overall pattern indicates limited recent inbreeding, punctuated by a small subset of animals with extensive autozygosity that should be monitored in future mating plans.

### 3.8. Analysis of the Family Structure of the Dengchuan Cattle Conservation Group

**Figure 7:**
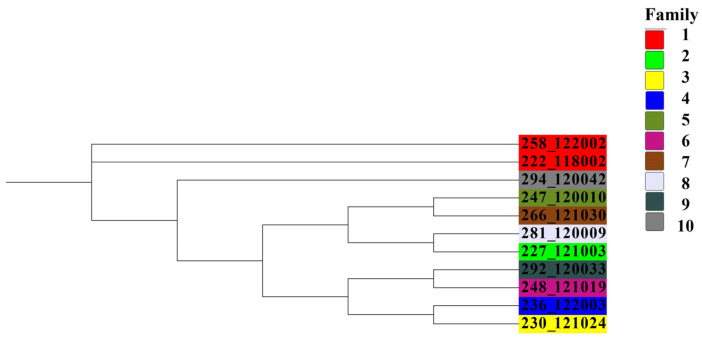
Results of cluster analysis of Dengchuan cattle bull groups.

**Figure 8:**
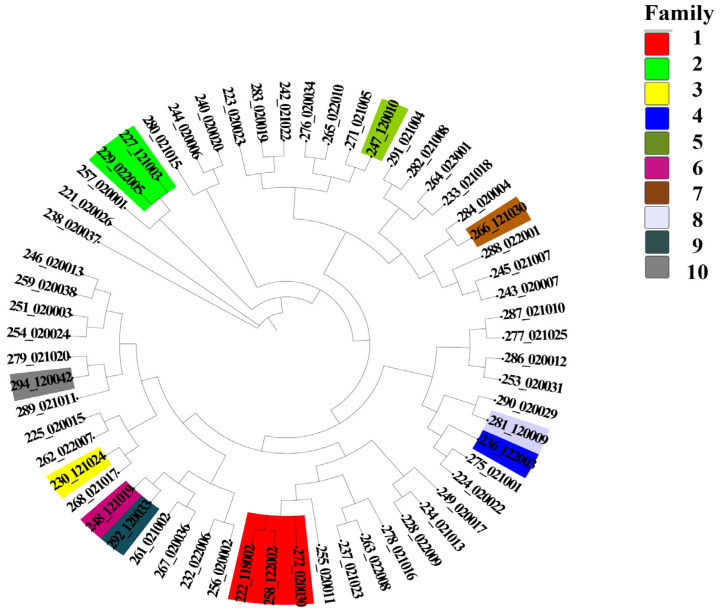
Cluster analysis of Dengchuan cattle. Different colors represent distinct families: Family 1 (Red), Family 2 (Lime Green), Family 3 (Yellow), Family 4 (Blue), Family 5 (Olive Green), Family 6 (Magenta/Fuchsia), Family 7 (Brown), Family 8 (Light Lavender/Off-white), Family 9 (Dark Teal/Dark Slate Gray), and Family 10 (Gray).

In the present study, 74 animals were genotyped, comprising 12 bulls and 62 cows. After the previously described screening approach (integrating PCA for extracting high-contribution loci and consistency analysis for identifying high-stability markers), 60 individuals were retained for family analysis. One bull was removed at this step as an outlier, leaving 11 bulls for downstream clustering. 11 bulls were clustered using the neighbor-joining method implemented in IQ-TREE software, resulting in the classification of these bulls into 10 distinct families. Notably, family line 2–10 contained only one bull (Figure 7). Given the significance of bulls within the conservation group, family lines were clustered based on a genomic kinship coefficient of ≥ 0.1 among bulls. Additionally, different family lines were categorized according to the kinship relationships between existing females and bulls. The results of the clustering analysis of the group samples are illustrated in Figure 8, revealing that no female samples could be classified into the corresponding families in family lineages 3, 4, 5, 6, 7, 8, 9, and 10. Furthermore, 46 cows within the group exhibited kinship coefficients of less than 0.1 with all bulls, indicating a distant relationship and leading to their classification into the ‘other’ category (Table 3).

## 4. Discussion

In terms of purity identification, this study employed PCA to extract the 100 SNP loci that contributed most significantly to PC1. This was combined with allele consistency analysis using PLINK (major allele frequency ≥ 0.8) to ultimately identify 61 highly conserved SNP markers. These loci demonstrate highly consistent major allele frequency distributions within the conservation population, with some nearing fixation, effectively reflecting the primary genetic background of the population. In contrast, Huan Wang [13] screened 3238 high-quality SNPs in a study of Hainan cattle by controlling for sequencing depth, missing rate, minor allele frequency, distribution uniformity, and functional site priority. This was further combined with 2939 functional sites from literature and databases to develop a 5K GBTS liquid chip. Compared to the current study, the purity identification strategy proposed here can yield a core set of loci that reflect the genetic characteristics of the population at a lower economic cost, offering insights for the development of a low-density SNP chip specific to Dengchuan cattle. Furthermore, the results indicated that most individuals exhibited high consistency with this set of SNPs, while a few individuals showed discrepancies with the major allele, suggesting a potential background of exogenous hybridization. This finding serves as a caution for the conservation of Dengchuan cattle, indicating that dynamic monitoring and optimization should be strengthened in future core germplasm management.

The effective population size (Ne) is a crucial parameter for evaluating genetic diversity within livestock populations and its evolution over time. When pedigree information is unavailable, recent Ne can be inferred from genome-wide linkage disequilibrium (LD) patterns derived from SNP data [14]. In population genetics, the strength of genetic drift—and thus the stability of allele frequencies—is determined by population size (and therefore by Ne): smaller populations experience stronger drift, increasing stochastic changes in allele frequencies, whereas larger populations buffer drift and help maintain polymorphism. Importantly, inbreeding per se does not change allele frequencies; rather, it increases homozygosity, which can unmask recessive deleterious alleles and lead to inbreeding depression. By contrast, genetic drift in small populations does change allele frequencies and can, by chance, fix or lose alleles over time. It is widely acknowledged that when Ne falls below 50, both drift and inbreeding become substantial, elevating the risk of inbreeding depression and accelerating the loss of genetic diversity [15,16]. Sudrajad [17] estimated effective population sizes (Ne) of 151 and 96 for Balinese cattle sourced from breeding centers (48 samples) and individual farms (54 samples), respectively, utilizing 50K SNP array data. He hypothesized that a livestock selection program in breeding centers is more conducive to maintaining genetic diversity in Balinese cattle. The Ne value of 2.293 for Dengchuan cattle is comparable to the Ne value of 2.6 reported by Pingping Wang [18]. Wang suggested that the lower Ne value may be attributed to the limited population size of Dengchuan cattle or the quantitative constraints of the sample utilized in this study, which comprised only 100 individuals. This assertion is further supported by the findings of Wang Hailong [19], lower Ne value and higher inbreeding increment would reduce the genetic diversity of the conserved population.

The observed heterozygosity ((Ho) reflects the actual proportion of heterozygous individuals in a population, while the expected heterozygosity (He) represents theoretical heterozygosity under Hardy–Weinberg equilibrium, serving as a marker of genetic diversity independent of selection, genetic drift, and other factors [20,21]. The Ho of Dengchuan cattle was 0.300, which is higher than the 0.297 reported for JuanShan cattle by Senczuk [22] et al. However, it is lower than the Ho in Italian Holstein cattle (0.342) and Swiss Brown cattle (0.320). The He of Dengchuan cattle was 0.326, which is higher than that of semi-wild Maremma [23] cattle (0.261); but lower than that of Egyptian Baladi [24] cattle (0.75). It is comparable to the heterozygosity of Dabie Mountain cattle (0.330), Wenshan cattle (0.330), and Zhaotong cattle (0.330) [25], all of which are indigenous breeds in southern China. A paired *t*-test confirmed a highly significant difference between Ho and He (*p* < 0.001), indicating that the observed heterozygosity is lower than the Hardy–Weinberg expectation. This heterozygote deficit, consistent with patterns reported in other indigenous breeds, is most plausibly explained by low-level inbreeding and/or population substructure (Wahlund effect), with possible contributions from selection at specific loci or null alleles. While genetic drift shapes allele-frequency trajectories and overall diversity in small populations, the immediate deviation between Ho and He in this conservation population is more directly attributable to departures from random mating and selection rather than drift perse.

MAF, or Minor Allele Frequency, refers to the frequency at which the less common alleles (also known as minor alleles) occur within a genetic locus. It serves as a crucial indicator of population genetic diversity, illustrating the distribution of alleles within a gene locus [26]. The MAF serves as a crucial indicator of genetic diversity within a population. A large MAF, approaching 0.5, suggests that the frequencies of the two alleles at a given gene locus are nearly equal, reflecting high genetic diversity. Conversely, a small MAF, nearing 0, indicates a dominant allele with a low frequency of the minor allele, which may suggest the presence of genetic drift or a bottleneck effect. In Matukumalli’s study [27], the MAF for cattle ranged from 0.24 to 0.31, while the MAF for Dengchuan cattle was reported at 0.239 ± 0.1370, closely aligning with the median value of 0.25. This finding indicates a moderate overall frequency of minor alleles within the population, suggesting that the population maintains a certain level of genetic diversity. It is important to note that while the observed MAF distribution is consistent with the effects of genetic drift in a small population, it is also likely influenced by an accumulation of recent, rare mutations, as you wisely pointed out. A formal Site Frequency Spectrum (SFS) analysis, which we propose as a valuable future step, would be essential for disentangling these distinct evolutionary forces.

PIC [28] is a critical index for assessing the polymorphism of a marker locus, such as a microsatellite marker or a SNP locus. It quantifies the contribution of the marker locus to the genetic diversity within a population. The calculation of PIC is based on the number of alleles present and their respective frequencies, thereby reflecting the level of polymorphism at the marker locus. A higher PIC value indicates greater polymorphism and enhanced genetic diversity. Specifically, a PIC value of less than 0.25 signifies low polymorphism, suggesting that the locus exhibits limited polymorphism and contributes minimally to genetic diversity. A PIC value between 0.25 and 0.5 indicates medium polymorphism, demonstrating a moderate degree of polymorphism. Conversely, a PIC value exceeding 0.5 denotes high polymorphism, indicating substantial genetic diversity and the potential for the locus to serve as an effective genetic marker. Mao [29] assessed the genetic diversity of Liangshan cattle utilizing the RAD-seq method and discovered that the polymorphic information content (PIC) value for cattle in the Liangshan region was 0.183. WeiWang [30] employed the RAD-seq method to determine that the average PIC of six breeds of Chuan cattle was 0.1555. Furthermore, the average PIC of Deng Chuan cattle was found to be 0.261 ± 0.101, indicating that, overall, the polymorphism of the loci was at a medium level and that the populations exhibited a certain degree of genetic diversity.

The SHI, also known as the Shannon–Wiener index, has been widely employed in various studies to estimate genetic diversity. It serves to describe variation across multiple levels of genetic organization, ranging from single-nucleotide polymorphisms (SNPs) to entire species or larger taxonomic units, and even ecosystems [31]. In Ruijun Wang’s article [32], the SHI averaged 0.530, concluding that the conserved populations of the IMCGs exhibited moderate polymorphism. The mean SHI value for Dengchuan cattle was 0.707, indicating high genetic diversity [33]. The fixation index (F) is a central tool for resolving the genetic structure of populations, revealing the effects of evolutionary forces such as inbreeding, migration, and drift on populations by quantifying the deviations and covariances of gene frequencies. The F values for Dengchuan cattle ranged from −0.193 to 0.384, with an average of 0.071 ± 0.122(SD). The mean Fi value for the Dengchuan cattle population was close to 0 but slightly positive, suggesting the potential presence of some blood duplications despite preservation efforts. The results of the SHI and Fi indicate that the genetic structure of Dengchuan cattle is relatively healthy; the preservation strategy appears to be initially effective, and there is no urgent need to introduce exogenous pedigrees at this time. However, greater attention should be directed towards the conserved population to prevent the rise in inbreeding in the future.

ROH refer to contiguous regions of the genome where all loci are homozygous, meaning that both alleles are identical. Typically identified through genomic analysis, ROH play a critical role in genetic studies by providing insights into the genetic background, degree of inbreeding, and selection signals of individuals or populations [34].

1. Overall, a total of 1770 ROHs were detected in 74 Dengchuan cattle, yielding an average of 29.50 ± 12.43(SD) ROHs per individual. This finding indicates significant variability in the presence of pure fragments among individuals within the population.
2. The study demonstrated that inbreeding leads to an increase in the length of ROH fragments [35]. The length of single ROH in the population predominantly ranged from 0 to 5 Mb, accounting for 71.1% of the total. This finding aligns with the results reported by Xu et al. [36]. which indicated that most ROH lengths among eight cattle breeds in China were also within 5 Mb. Conversely, the occurrence of single ROH lengths ranging from 15 to 20 Mb was minimal, constituting only 3.50% of the total. This suggests that the population is primarily characterized by short fragments resulting from distant inbreeding, with relatively few recent inbreeding events.
3. At the chromosome level, the distribution of ROH across the 29 autosomes was uneven. Chromosome 8 exhibited the highest number of ROH fragments, totaling 115, while chromosome 27 had the lowest, with only 23 fragments. This disparity in the accumulation of pure fragments across different chromosomes may be attributed to genetic drift or selective pressures acting on specific chromosomes, such as those associated with quantitative trait loci (QTL) related to meat quality, body size, and reproduction. These factors are likely intensified through repeated selection during conservation or breeding processes.
4. The mean value of the F_ROH_ group was 0.0928 ± 0.1054, which is higher than the inbreeding coefficients reported for 11 Polish cattle breeds studied by Szmatoła [37]. Specifically, the coefficients for Polish Red and White Friesian cattle, Limousin cattle, Charolais cattle, Simmental cattle, Polish Red cattle, Polish Red and White cattle, and Polish Black and White cattle were 0.042, 0.059, 0.065, 0.068, 0.053, 0.042, and 0.054, respectively. Additionally, Holstein-Friesian Red and White cattle had an inbreeding coefficient of 0.087, while Hereford cattle, Holstein-Friesian Black and White cattle, and Montbéliard cattle had coefficients of 0.151, 0.118, and 0.108, respectively. These findings indicate that the inbreeding level of the Dengchuan cattle population is low; however, it is essential to introduce new purebred bloodlines to enhance population size and genetic diversity. Consistently, the mean F based on Hardy–Weinberg equilibrium was 0.071 ± 0.122 (SD), also significantly greater than zero (*p* < 0.01). The similarity of F_ROH_ and F results provides complementary evidence for the existence of a low but significant level of inbreeding in the conservation population. The highest percentage of individuals with F_ROH_ values ranging from 0 to 0.06 reflects a generally low overall inbreeding level within the population, although significant variation exists among individuals. Further analysis of sex differences revealed that the F_ROH_ was 0.119 ± 0.107(SD) for bulls and 0.087 ± 0.105(SD) for females, indicating a slightly higher level of inbreeding among bulls. This may be attributed to their more frequent use as primary breeding individuals and the concentration of breeding sources. The results of the NJ cluster analysis indicated that the 11 bulls could be classified into 10 lineages, with 9 lineages (lineages 2 to 10) comprising a small number of bulls, leading to an unbalanced structure. Therefore, careful attention should be paid to the selection and breeding of progeny in the future to prevent loss of pedigree. The 46 females classified under the category of ‘Others’ exhibited a kinship coefficient of less than 0.1 with all the bulls, allowing them to be mated with any bulls within the group.

## 5. Conclusions

In this study, we utilized the bovine 100K SNP microarray to analyze, at the molecular level, the top 100 SNP loci contributing most significantly to PC1 through principal component analysis (PCA). Based on this analysis, we identified 61 loci with a high degree of concordance, characterized by a principal allele frequency of ≥ 0.80. Utilizing these 61 loci as the foundation, we conducted genotypic concordance analyses across individual populations, ultimately identifying 60 representative Dengchuan cattle with high genetic purity. The total number of SNP loci for the 74 Dengchuan cattle was 95,256, and the same total was observed for the 60 Dengchuan cattle, indicating that these 60 individuals are indeed representative. The results revealed that the overall genetic diversity of the conserved population was moderate (He = 0.326), the level of inbreeding was low (average F_ROH_ = 0.0928), yet the effective population size was small (Ne = 2.293). Additionally, there was a notable imbalance in the lineage structure of the bulls, suggesting that further optimization of the breeding strategy is necessary to expand the genetic base of the population. The 61 highly consistent SNP loci established in this study serve as an effective tool for purity assessment, core germplasm screening, and long-term genomic monitoring of Dengchuan cattle. They provide both theoretical and technical support for the scientific conservation and sustainable utilization of local high-quality dairy cattle breeds.

## Figures and Tables

**Table 1:** Statistical results of SNP quality control in 74 Dengchuan cattle.

Quality Control Criteria	Number of Markers of SNPs
Total number of markers	95,256
Markers on sex chromosomes (X, Y) or with unassigned chromosomal location (0)	4907
SNP detection rate < 0.90	8478
Markers with *p* < 10^−6^ in Hardy–Weinberg balance test	71
Markers with minor allele frequency < 0.01	3340
Number of SNPs passing quality control	78,460

**Table 2:** Statistical results of SNP quality control of 60 Dengchuan cattle.

Quality Control Standard	Number of SNPs Markers
Total number of markers	95,256
Markers on sex chromosomes (X, Y) or with unassigned chromosomal location (0)	4907
SNP detection rate < 0.90	8242
Markers with *p* < 10^−6^ on Hardy–Weinberg balance test	50
Markers with minor allele frequency < 0.01	5111
Number of SNPs passing quality control	76,946

**Table 3:** Family classification of Dengchuan cattle.

Family Name	Sex	Number	Individual Number
Family 1	Male	2	258_122002
			222_118002
	Female	1	272_020030
Family 2	Male	1	227_121003
	Female	1	229_022005
Family 3	Male	1	230_121024
	Female		
Family 4	Male	1	236_122003
	Female		
Family 5	Male	1	247_120010
	Female	1	
Family 6	Male	1	248_121019
	Female		
Family 7	Male	1	266_121030
	Female		
Family 8	Male	1	281_120009
	Female		
Family 9	Male	1	292_120033
	Female		
Family 10	Male	1	294_120042
	Female		
Other	Female	46	

## Data Availability

https://zenodo.org/records/17239145?token=eyJhbGciOiJIUzUxMiJ9.eyJpZCI6IjBmZjQ0NTQ3LTM1MmYtNDQyNC1iM2JlLTBhMWM0ZDc0M2Q3NyIsImRhdGEiOnt9LCJyYW5kb20iOiJiODc1Y2IyYjVlYzk4MThhZDUyOTQ3YTM1YzM2Y2QwZCJ9.egKECoyfxFnEtGXkp9tikVGrFFBw3BM0s1SChhrtlXtcpTNTWPkldGCLP97F75Ec2ZeIdUiuWI9V0uRFvmPvdw (accessed on 1 October 2025).

## Supplementary Materials

The following supporting information can be downloaded at: https://www.mdpi.com/article/10.3390/ani15202937/s1, Table S1: Individual Inbreeding Coefficient Metrics, Table S2: SNP Diversity Metrics, Table S3: High contributing loci in 100 PC1s, Table S4: Primary and secondary alleles at 61 loci, Table S5: The top 60 Dengchuan cattle screened out.
